# Immunological studies on new rabbit paternal lines with different potentials for growth rate and resilience: pathways towards healthier animals

**DOI:** 10.1186/s13567-025-01664-z

**Published:** 2025-12-12

**Authors:** Catarina Peixoto-Gonçalves, Eugenio Martínez-Paredes, Elena Moreno Grúa, Carmen Martínez Seijas, David Viana, Alberto Arnau Bonachera, Luis Ródenas, María Cambra-López, Enrique Blas, Juan José Pascual, Juan Manuel Corpa

**Affiliations:** 1 Institute for Animal Science and Technology, Universitat Politècnica de València, Camino de Vera, s/n, 46022 Valencia, Spain; 2 Biomedical Research Institute (PASAPTA-Pathology Group), Facultad de Veterinaria, Universidad Cardenal Herrera-CEU, CEU Universities, C/Tirant Lo Blanc 7, 46115 Valencia, Spain; 3 Department of Animal Production and Health, Veterinary Public Health and Food Science and Technology, Veterinary School, Universidad Cardenal Herrera-CEU, 46115 Valencia, Spain

**Keywords:** Immunology, genetic lines, flow cytometry, rabbit, stayability

## Abstract

This study evaluated leukocyte and immune parameters in female rabbits and their kits from three genetic lines (R, RF, and RFLP) during their first reproductive cycle. The R line was selected for growth rate over 37 generations, the RF line was derived from elite R animals, and the RFLP was obtained by backcrossing the RF line with a maternal line (LP). In females, the total lymphocyte percentage decreased from the first AI to weaning (−15.8 percentage points; *p* < 0.001; MIXED model); granulocytes peaked at weaning (+12.4 percentage points greater than the AI and +4.4 percentage points greater than parturition; *p* < 0.01; MIXED model). The CD4^+^ /CD8^+^ ratio increased 53.4% (*p* < 0.001; MIXED model), and the haptoglobin concentration doubled (*p* < 0.001; MIXED model). RF and RFLP had higher total lymphocyte percentages than R did (+6.4 and +4.6 percentage points; *p* < 0.05; MIXED model). R females had lower CD4^+^ percentages and CD4^+^ /CD8^+^ ratios than RLFP females did (−3.4 percentage points and −24.4%; *p* < 0.05; MIXED model) but higher monocyte and granulocyte percentages (on average +0.9 and +4.1 percentage points; *p* < 0.05; MIXED model). At weaning, R kits had fewer T lymphocytes and CD4^+^ T lymphocytes than RF kits did (−8.1 and −6.1 percentage points; *p* < 0.05; GLM) and lower CD25^+^ and phagocytosis of heterophils than RFLP kits did (−0.8 and −1.2 percentage points; *p* < 0.05; GLM). The results suggest that creating a paternal line via elite animals (RFs) and backcrossing it with a robust line could improve immune status and stayability, offering insights into the impacts of rabbit breeding on immune health and reproductive success.

## Introduction

Traditionally, animal breeding has focused on selecting traits such as growth rate and milk production, with little consideration of immune function. Despite the apparent benefits of high production efficiency, animals in a population that has undergone selective breeding for high production may also face various behavioural, physiological, and immunological challenges, primarily related to susceptibility to recurrent pathogens and environmental stress factors, where rabbits are vulnerable to heat [1]. For example, in chickens, intensive selection for production and feed efficiency seems to have come at the expense of immunity, as evidenced by low adaptative immune responses and high vulnerability to illness [2]. The intensive selection for high milk yield in dairy cattle could affect heat stress tolerance, negatively impacting female health and milk production [3]. Compared with those from a more robust maternal line, young rabbits from paternal lines selected for productive traits presented a greater incidence of digestive disorders during the growing period [4]. In addition, females from a paternal line display lower immune adaptability to reproductive challenges than females selected for high robustness [5].

A previous study discussed the potential benefits of selecting animals for a strong immune response to improve overall animal health and food safety [6]. The authors argued that selecting for a more advantageous immune response could increase disease resistance and reduce the degree of dependence on antimicrobials. In addition, a recent study using females from a paternal rabbit line (the R line) reported that selective breeding for increased growth rates did not impair immune responses to bacterial infection, suggesting that a relatively high growth rate does not necessarily compromise immune competence [7]. These findings highlight the complex relationship between growth performance and immune competence and the need to further investigate potential trade-offs.

Our research group considered two strategies to improve rabbit health and reproduction and reduce antimicrobial dependence in a paternal line selected for average daily gain (ADG). The R paternal line served as the control group, representing the baseline genetic background, and two new genetic lines were created from these animals. The first genetic line (RF) was formed by selectively breeding elite animals from the R line on the basis of their high growth rate, specifically selecting individuals whose ADG was greater than 60 g/d. This approach aimed to establish a new genetic line with enhanced growth performance. On the other hand, to improve the RF line’s overall robustness and disease resistance, we introduced genetics from a long-living maternal line (LP) through backcrossing, creating the second genetic line (RFLP). Previous results suggest that the RF line produced females with better reproductive performance during the first reproductive cycle and that the reproductive performance of RFLP females was more consistent throughout their reproductive life [8]. Furthermore, young growing rabbits from the RFLP line presented lower mortality during the growth period [9]. We hypothesise that the founding process carried out with the new genetic lines improved their immune status. To verify this hypothesis, we aimed to identify possible differences in the composition and functionality of the immune system between the R, RF and RFLP lines by examining immune cell populations and haematological profiles of rabbit females and their kits.

## Materials and methods

### Animals and experimental procedure

A total of 122 female rabbits from 3 paternal lines were used for this experiment. The animals belonged to lines R (*n* = 40), RF (*n* = 37) and RFLP (*n* = 45), which were developed by the Animal Breeding Group of the Universitat Politècnia de València in Spain. The R line is a paternal line selected for its growth rate during the growing period over 37 generations [10]. The RF line was founded by selecting a population of elite R animals from different genetic selection centres, which exhibited a high growth rate during the growing period (more than 60 g/d; ADG of the final population: 61.94 g/d). The RFLP line was created by backcrossing males from the RF line with females from the LP maternal line, generating animals with 7/8 RF and 1/8 LP genetics. The LP line was founded on productive longevity criteria (a minimum of 25 parturitions and 7.5 kits born alive) [11].

The animals were kept in a controlled environment with a conventional ventilation system and a photoperiod of 16 h of light and 8 h of darkness. The female rabbits were randomly selected at 9 weeks of age and housed in individual cages for reproduction (700 × 500 × 320 mm) until the end of the trial at first weaning.

The female rabbits were fed ad libitum with two different commercial diets throughout the experiment. Until the first parturition, the females were fed a diet for growing rabbits (158 g crude protein, 25 g ether extract and 231 g crude fibre per kg of feed) and then a diet for reproductive rabbit females (193 g crude protein, 40 g ether extract and 169 g crude fibre per kg of feed). Both commercial diets met the current recommendations for reproductive rabbit females [12]. The animals were artificially inseminated (AI) at 20 weeks of age. Gestation diagnosis was performed by manual palpation at 11 days post-AI. Non pregnant females were reinseminated 21 days later until a maximum of 3 attempts were made. After the 28^th^ day of gestation, an external nest equipped for the litter was attached (220 × 400 × 380 mm). At parturition, the number of kits per litter was standardised to 6. Litter size and weight were controlled at birth (total, alive and standardised) and weaning. The litters were weaned at 28 days post-partum (dpp). The mortality of the mothers and kits was recorded daily. The reproductive females received no antibiotics and were only vaccinated against rabbit haemorrhagic disease, starting at two months of age and then every eight months until the end of the experiment. The kits were neither vaccinated nor given antibiotics.

Blood samples from female rabbits were collected at the first AI, first parturition, and first weaning to evaluate the immune system’s baseline, monitor changes due to physiological stress, and assess immune responses during critical reproductive stages. These samples help elucidate the impact of genetic lines on immune health and reproductive success. In addition, blood samples were collected from three kits per litter at first weaning. These samples were mixed and processed as a single sample. For the analysis of haptoglobin and immunophenotype, tubes with EDTA were used, and for phagocytosis studies, acid-citrate dextrose-containing tubes were used. Blood samples were collected at approximately the same time (9:00 am to 10:00 am) to prevent diurnal variations in haematological parameters.

### Immunophenotypic characterisation of peripheral blood by flow cytometry

One millilitre of blood was lysed with 45 mL of ammonium chloride lysing solution to prepare the samples. The resulting leukocyte pellet was resuspended in 1 mL of Dulbecco’s phosphate-buffered saline (DPBS) (Sigma‒Aldrich^®^). Each animal’s cell suspension was then divided into six sample tubes. Primary monoclonal antibodies (Table 1) were added to the cell suspension according to the manufacturer’s instructions, and the mixture was incubated for 25 min at room temperature. The samples were washed, and secondary antibodies (rat anti-mouse IgG2ak or IgG2bk phycoerythrin [Nordic–MUbio] and goat anti-mouse IgM:R-phycoerythrin [Bio-Rad]) were added and incubated for 25 min at room temperature. The samples were then rewashed, and 1 mL of DPBS was added before analysis on the flow cytometer. The leukocyte (WBC) suspensions were analysed using a CytoFLEX S flow cytometer (Beckman Coulter, Indianapolis, IN, USA). For each sample, 50 000 cells were measured. First, gates were set to define the populations of lymphocytes, monocytes, and granulocytes. The combination of cell size, complexity, and leukocyte antigens was used to differentiate the granulocyte population from the lymphocyte and monocyte populations. The “lymphogate” phenotype was defined using the common leukocyte antigens CD14 and CD45, as previously described [13, 14], to determine the total percentage of lymphocytes, monocytes, and granulocytes. These gates were then used to identify the remaining subpopulations with the corresponding markers. From the total percentage of lymphocytes, the percentages of B-lymphocytes and T-lymphocytes were calculated as IgM^+^ and CD5^+^, respectively. Within the T lymphocyte population (CD5^+^), the percentages of T lymphocyte subsets were further identified as CD5^+^–CD4^+^, CD5^+^–CD8^+^ and CD5^+^–CD25^+^, as described previously [14].

**Table 1.** **Monoclonal antibodies used for flow cytometry**

Monoclonal antibody	Iso	Spec	Cell labelling	Clone	Refs.	Comp.
Mouse anti-rabbit T lymphocytes: FITC^1^	IgG1	CD5	T-cell	KEN-5	[42]	Abd Serotec
Mouse anti-rabbit α-pan B	IgM	IgM	B-cell	MRB143A	[32]	VMRD Inc
Mouse anti-rabbit CD4	IgG2a	CD4	T-cell subset	KEN-4	[42]	Abd Serotec
Mouse anti-rabbit α-CD8	IgG2a	CD8	T-cell subset	ISC27A	[32]	VMRD Inc
Mouse anti-rabbit CD25	IgG2b	CD25	Activated T cells	KEI-ALPHA1	[43]	Abd Serotec
Mouse anti-human CD14: FITC	IgG2a	CD14	Monocytes and granulocytes	TϋK4	[44]	Abd Serotec
Mouse anti-rabbit α-CD45	IgM	CD45	All leukocytes	ISC76A	[32]	VMRD Inc

^1^Clon KEN-5 recognises rabbit T lymphocytes and immunoprecipitates. This antibody recognises rabbit CD5 but does not bind to rabbit CD5 transfectants. Known rabbit CD5 antibodies also bind to most B lymphocytes, which are not labelled by this clone (information obtained from the datasheet).

Iso: isotype; Spec: specificity; Refs: references; Comp: company.

### Determination of haptoglobin

For the analysis of haptoglobin, the blood samples were centrifuged (3000 × *g*, 4 °C and 10 min), the plasma was separated, and the samples were stored at −40 °C for subsequent analysis. Haptoglobin was determined on the basis of its ability to bind to haemoglobin (Phase™, Tridelta Developments, Wicklow, Ireland). For its determination, an autoanalyzer, the ADVIA 1800^®^ Chemistry 53 System, was used (Siemens Medical Solutions, Tarrytown, NY 10591, USA). The intra- and interassay CVs were consistently less than 2.0% and 4.0%, respectively.

### Purification of polymorphonuclear leukocytes (PMNs) and macrophages

The purification of PMNs (also known as heterophils) and macrophages was performed according to previously described protocols [15–17]. Rabbit blood was collected with acid-citrate dextrose, and Hetastarch (Sigma‒Aldrich) was added to separate the PMNs by causing erythrocyte sedimentation. Neutrophils were isolated using a gradient with Histopaque 1077 (Sigma‒Aldrich). For monocytes, peripheral blood mononuclear cells (PBMCs) were isolated with Lymphoprep (Palex, Madrid, Spain). After density gradient separation, the PBMCs were washed and cultured in RPMI-1640 medium supplemented with 10% foetal bovine serum (FBS) and 1% penicillin/streptomycin (Thermo Fisher, Massachusetts, USA). Nonadherent cells were removed after 2 h of incubation. The macrophages were polarised in vitro by culturing adherent cells for 6 days in RPMI-1640 medium supplemented with 10% FBS, 1% penicillin/streptomycin, and recombinant human GM-CSF. Cell count and viability were determined via a hemacytometer and trypan blue.

### Phagocytosis of PMNs and macrophages

To assess the phagocytosis of macrophages and PMNs, fluorescent yellow‒green latex beads with a mean particle size of 2.0 μm (Sigma‒Aldrich^®^) were used. An inoculum of beads was prepared by adding 10 times more latex beads than cells (10^6^ cells/mL). For both protocols, three replicates and a negative control (without beads) were performed for each animal. Animals with less than 5% cell confluence in the wells were excluded from the study. Phagocytosis was allowed to proceed for 30 min at 37 °C before being stopped with ice. For the phagocytosis of macrophages, cells were collected from the wells with porcine pancreas trypsin solution (1x) (Sigma‒Aldrich^®^) and then incubated with the monoclonal antibody CD11b-PE (Thermo Fisher^®^) according to the manufacturer’s instructions. For phagocytosis of the PMN, the cells were labelled with the monoclonal antibody CD11b-PE (Thermo Fisher^®^) according to the manufacturer’s instructions and fixed with paraformaldehyde (4%). Finally, the cells were resuspended in 1 mL of DPBS and transferred to cytometer tubes for analysis using a CytoFLEX S flow cytometer (Beckman Coulter, Indianapolis, IN, USA). All cells marked with the CD11b-PE antibody were considered part of the granulocyte or macrophage population. Depending on the protocol, the cells that had phagocytosed were identified by the FITC fluorescence of the latex beads, whereas the cells that had not phagocytosed did not show this fluorescence. The kits’ blood samples were not used to determine the degree of macrophage phagocytosis.

### Statistical analysis

When asymmetrical distributions were found, the original data were transformed logarithmically. The effect of the physiological state on the leukocyte percentage in females was analysed via a MIXED model of SAS (Statistical Analysis System) for repeated measures. The model included the genetic type, physiological state, interaction, and batch as fixed effects and the animals as permanent effects. The leukocyte percentage of kits at weaning was analysed via a GLM from SAS, which included only the genetic type as a fixed effect. Pearson’s correlation coefficients (ρ) were obtained via PROC CORR in SAS to test the relationships between the percentages of leukocytes in females and kits. Differences were considered significant at *p* < 0.05.

## Results

Table 2 shows the leukocyte percentage of the females’ blood from the first artificial insemination to the first weaning. The effect of the physiological state was significant for all the measured parameters (*p* < 0.001). The total lymphocyte percentage drastically decreased from the first AI to weaning (−15.8 percentage points). Similarly, the percentages of T lymphocytes and CD4^+^, CD8^+^, and CD25^+^ T lymphocytes also decreased from the first AI to weaning (−7.7, −3.5, −4.1 and −1.4 percentage points, respectively). The percentage of B lymphocytes varied across the reproductive cycle, increasing from the first AI to parturition (+3.2 percentage points) and subsequently decreasing from parturition to weaning (−6.2 percentage points). In contrast, the monocyte percentage reached its lowest value at parturition, followed by an increase at weaning (+1.5 percentage points; *p* < 0.001). The granulocyte percentage significantly differed among the three time points, reaching the highest value at weaning (12.4 percentage points higher than that at AI and 4.4 higher than that at postpartum). The CD4^+^/CD8^+^ ratio increased by 53.41% from the first AI to weaning. The haptoglobin concentration was doubled from the first AI to weaning. The phagocytosis of macrophages significantly decreased from the first AI to weaning (on av. −17.6 percentage points; *p* < 0.001). However, the phagocytosis of heterophils increased from the first AI to weaning (on av. +6.3 percentage points; *p* < 0.05).

**Table 2.** **Effect of genetic type and physiological state on the percentage of leukocytes in the blood of rabbit females**

	Genetic type				State				*P* value	
	R	RF	RFLP	SEM	AI	P	W	SEM	GT	S
n	40	37	45		122	122	122			
Total lymphocytes	33.47^a^	39.89^b^	38.05^b^	1.598	42.70^b^	41.79^b^	26.92^a^	1.531	0.016	< 0.0001
B -Lymphocytes	17.00	16.45	15.55	1.089	16.27^a^	19.46^b^	13.28^a^	1.059	0.625	0.0007
T-Lymphocytes	35.40	36.76	37.56	1.166	39.84^b^	37.72^b^	32.16^a^	1.048	0.404	< 0.0001
CD4^+^	24.26^a^	25.97^ab^	27.67^b^	0.922	26.67^b^	28.08^b^	23.16^a^	0.830	0.032	0.0002
CD8^+^	8.178	8.304	7.599	0.428	10.48^b^	7.197^a^	6.406^a^	0.360	0.455	< 0.0001
CD25^+^	3.902	4.055	3.462	0.265	4.691^b^	3.449^a^	3.280^a^	0.275	0.262	0.0007
Monocytes^1^	5.535^b^	4.553^a^	4.636^a^	0.277	5.208^b^	4.078^a^	5.538^b^	0.256	0.023	0.0004
Granulocytes	32.26^b^	28.03^a^	28.35^a^	1.285	23.95^a^	28.35^b^	36.35^c^	1.233	0.038	< 0.0001
CD4^+^/CD8^+^	3.313^a^	3.717^ab^	4.385^b^	0.243	2.756^a^	4.436^b^	4.228^b^	0.218	0.007	< 0.0001
Haptoglobin (log_10_ 10^9^/L)	−0.478	−0.441	−0.456	0.058	−0.701^a^	−0.687^a^	−0.399^b^	0.081	0.899	< 0.0001
Phagocytosis (%)										
Macrophages	67.88	67.13	62.82	2.30	77.73^b^	60.10^a^	60.01^a^	2.33	0.256	< 0.0001
Heterophils	43.84	43.98	44.21	1.85	39.77^a^	46.20^b^	46.05^b^	2.09	0.797	0.119

R: paternal line selected by growth rate; RF: new paternal line founded by selecting elite animals from the R line; RFLP: new paternal line founded by backcrossing the lines RF and a maternal line called LP.

SEM: pooled standard error of means; AI: artificial insemination; P: partum; W: weaning; GT: genetic type; S: physiological state.

^a,b,c^Means in a row not sharing superscripts were significantly different (*P* < 0.05).

^1^Interaction GT*S.

**Figure 1:**
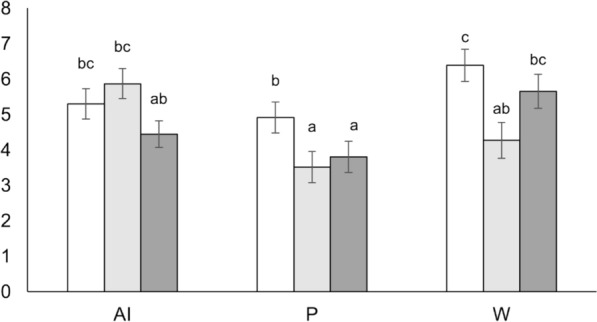
**Effects of genetic type (R, RF and RFLP) on the percentage of monocytes in female rabbits from the first AI to weaning**. AI: artificial insemination; P: parturition; W: weaning. Means not sharing superscripts are significantly different (*P* < 0.05).

The effect of genetic type on the leukocyte percentage in the blood of females was significant for only some of the evaluated parameters. The RF and RFLP lines presented a greater total lymphocyte percentage than did the R line (+6.4 and +4.6 percentage points; *p* < 0.05). The CD4^+^ and CD4^+^/CD8^+^ ratios for the R line were significantly lower than those for the RLFP line (−3.4 percentage points and −24.4%, respectively; *p* < 0.05). However, the monocyte and granulocyte percentage values were significantly greater for the R line than for both the RF and RFLP lines (on av. +0.9 and +4.1 percentage points, respectively; *p* < 0.05). For all the parameters, the monocyte percentage was the only one that showed an interaction between the genetic type and physiological state. As shown in Figure 1, there were no significant differences among the three genetic types at first AI. However, the R line had a higher monocyte percentage than both RF and RFLP at first parturition (on av. +1.3 percentage points; *p* < 0.05) and higher than that of RF at first weaning (+2.1 percentage points; *p* < 0.05). In general, there were no significant differences in either the leukocyte percentage or the phagocytosis percentage between RF and RFLP females. With respect to the concentration of haptoglobin in the plasma of female rabbits, there were no significant differences among the three genetic lines during the first reproductive cycle.

Table 3 shows the leukocyte percentage of the kits’ blood at first weaning. The percentages of T lymphocytes and CD4^+^ cells in the R kits were significantly lower than those in the RF kits (−8.1 and −6.1 percentage points, respectively; *p* < 0.05). In addition, kits from the R line presented the lowest values for the percentage of CD25^+^ cells (on av. −0.8 percentage points; *p* < 0.05). With respect to the percentage of phagocytosis of heterophils, the R-line kits presented significantly lower values than did the RFLP-line kits (−1.2 percentage points; *p* < 0.05), whereas the RF-line kits presented intermediate values.

**Table 3.** **Effect of the genetic type on the percentage of leukocytes in weaned rabbits**

	Genetic type				*P* value
	R	RF	RFLP	SEM	GT
No. of litters	19	15	18		
Total lymphocytes	1.278	1.528	1.043	0.144	0.082
B-Lymphocytes	4.912	4.946	5.057	0.570	0.982
T-Lymphocytes	15.50^a^	23.64^b^	20.54^ab^	2.176	0.036
CD4^+^	9.882^a^	15.98^b^	13.76^ab^	1.458	0.016
CD8^+^	4.904	7.049	6.174	0.685	0.096
CD25^+^	0.736^a^	1.420^b^	1.502^b^	0.198	0.012
Monocytes	3.589	3.483	3.839	0.419	0.835
Granulocytes	14.05	15.92	17.81	2.442	0.534
CD4^+^/CD8^+^	2.191	2.360	2.288	0.135	0.679
Phagocytosis (%)					
Heterophils	1.812^a^	2.874^ab^	3.010^b^	0.454	0.121

R: paternal line selected by growth rate; RF: new paternal line founded by selecting elite animals from the R line; RFLP: new paternal line founded by backcrossing the lines RF and a maternal line called LP.

*n* number of records; SEM: pooled standard error of means; GT: genetic type.

^a,b^Means in a row not sharing superscripts were significantly different (*P* < 0.05).

**Figure 2:**
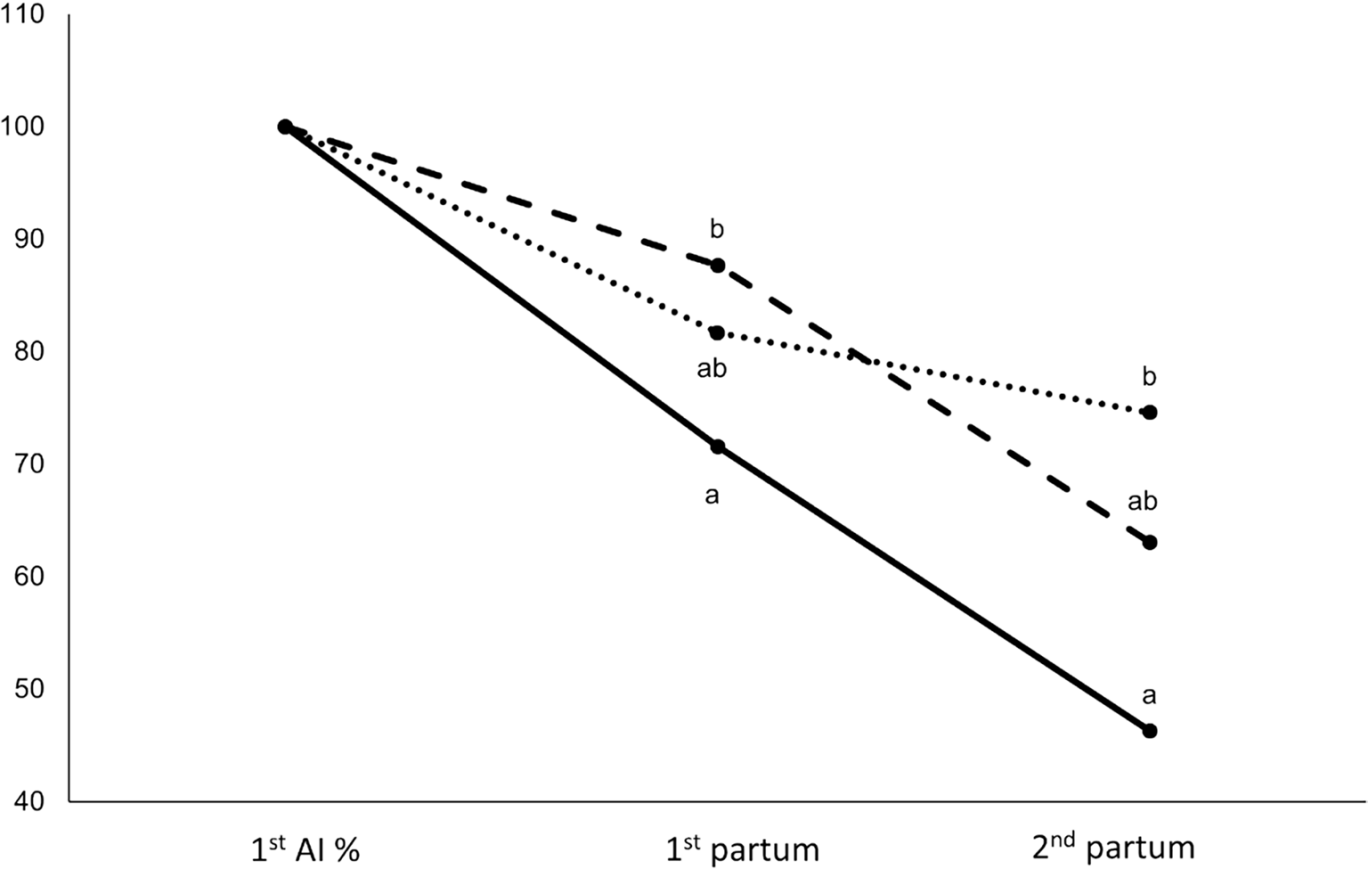
**Evolution of female survival (%) from the first AI to the second parturition (R; RF; RFLP).** Means in a physiological stage not sharing superscripts were significantly different (*P* < 0.05).

Figure 2 shows the females’ survival from the first AI to the second parturition. At the first parturition, the proportion of females from the R line significantly differed from that from the RF line (−16.0 percentage points; *p* < 0.05). At the second parturition, the proportion of the R line population was lower than that of the RFLP population (−24.0 percentage points; *p* < 0.05), with RF females having intermediate values. The proportion of females from the R line showed the greatest decline among the three lines (53.7 percentage points).

Table 4 presents Pearson correlation coefficients demonstrating associations between the percentage of leukocytes in females and their kits at weaning. Specifically, an increase in the percentage of total lymphocytes in females corresponds to higher percentages of total lymphocytes, T lymphocytes, and CD4^+^ and CD8^+^ lymphocytes in their kits (+0.339, +0.370, +0.409 and +0.305, respectively; *p* < 0.05). Similarly, populations of granulocytes, CD8^+^ and CD4^+^/CD8^+^ cells from females and their kits were also correlated at weaning (+0.402, +0.297 and +0.412, respectively; *p* < 0.05). Furthermore, higher percentages of T lymphocytes and CD4^+^ T lymphocytes in females were associated with increased CD25^+^ T lymphocytes in their kits (+0.334 and +0.295, respectively; *p* < 0.05).

**Table 4.** **Pearson coefficients for the correlations between leukocyte populations of females and kits at weaning**.

	Females								
	Total Lymphocytes	Macrophages	Granulocytes	B-lymphocytes	T-lymphocytes	CD4^+^	CD8^+^	CD25^+^	CD4^+^/CD8^+^
Kits									
Total lymphocytes	0.339*	−0.138	−0.325	0.029	−0.009	−0.104	0.070	−0.089	−0.147
Macrophages	0.072	0.154	0.214	0.156	0.192	0.128	0.049	0.121	0.062
Granulocytes	−0.096	0.113	0.402**	0.069	−0.059	−0.184	0.099	0.214	−0.152
B-lymphocytes	0.053	−0.098	0.060	0.153	−0.026	−0.096	0.131	0.123	−0.203
T-lymphocytes	0.370**	0.071	0.038	0.031	0.212	0.005	0.206	0.177	−0.149
CD4^+^	0.409**	0.049	−0.015	0.023	0.217	0.031	0.180	0.179	−0.097
CD8^+^	0.305*	0.099	0.046	0.025	0.214	−0.035	0.297*	0.133	−0.277
CD25^+^	0.220	0.050	0.087	0.008	0.334*	0.295*	−0.055	0.238	0.219
CD4^+^/CD8^+^	0.222	−0.020	−0.242	−0.038	−0.129	0.021	−0.323	0.003	0.412**

*n* number of records; *n* = 52

*p < 0.05; **p < 0.01.

## Discussion

Haematological studies have shed light on aspects such as the immune system’s activation level, the short- and long-term effects of poor husbandry practices, potential illnesses, and genetic predispositions [18]. In rabbits, haematological and biochemical parameters are influenced by stress, age, sex, breed, and circadian rhythms [19]. Since the immune system of rabbits might subsequently respond to multiple factors throughout their life, the first AI is the ideal moment to evaluate the immune system in relation to its genetic type [20]. Therefore, the sample taken at first AI was assumed to be a control to compare the changes throughout the animals’ lives.

The energy deficit resulting from the overlap between gestation and lactation causes significant body mobilisation and a decrease in reproductive performance. This problem is particularly pronounced in primiparous rabbits, as their feed intake capacity is not yet fully developed, and their growth remains incomplete. This is one of the main reasons why, in female rabbits, the first weaning and second parturition are critical moments with high culling risks [21–23]. Stress caused by excessive production reduces the immune response (immunological stress) and increases the risk of disease. For R females, the variations in the leukocyte profile were analysed depending on the reproductive stage of the animal, suggesting that female rabbits face greater immunological stress during the first weaning, where they show higher levels of resource mobilisation to compensate for the energy deficit [5]. In a recent study comparing two different generations of the R line, the concentrations of total lymphocytes and the main lymphocyte populations (T lymphocytes, CD4^+^, CD8^+^ and CD25^+^) decreased from the first AI to weaning [7]. Similarly, our results revealed a decrease in the percentage of total lymphocytes and the main lymphocyte populations at weaning. Since adaptive immunity is suppressed during gestation to prevent foetus rejection, a decrease in the percentage of the main leukocyte populations is expected [24]. Additionally, physiological stress related to a high resource requirement between competing functions (growth, lactation and gestation) may induce general leukopenia with lymphopenia and an increase in granulocytes [19]. In our study, although absolute values were not analysed, significant changes in leukocyte percentages were detected during the reproductive cycle in female rabbits. Specifically, the total lymphocyte percentage decreased from the first artificial insemination (AI) to weaning, whereas the granulocyte percentage increased significantly during the same period. These findings align with the expected immunological adjustments during gestation and lactation. Adult rabbit females with longer lactation periods (later weaning) have lower total lymphocyte counts and lymphocyte subtypes than females weaning their kits earlier [14]. Furthermore, an increase in granulocytes with a lower number of lymphocytes is related to an increase in immunological stress and is considered a biological indicator that predicts future health issues in animals. Chronic elevation of granulocytes can lead to chronic inflammation, immune system imbalance and increased susceptibility to infections [25]. A decrease in lymphocyte populations directly impacts the adaptive responses of animals to illnesses [26]. These cells initiate, maintain, and control the immune response [27]. Therefore, a pattern of recurrent infection appears when there is a deficit of T cells [28].

A stress-related decrease in phagocytic activity has been described [29, 30]. In this work, the decrease in macrophage activity from the first AI to weaning might also be an indicator of stress at this particular moment. However, the increase in the granulocyte percentage and heterophil activity observed in this work might suggest some improvement in innate immunity [31]. An increase in the innate immune response could constitute an upregulation strategy to compensate for the decreased adaptive immune function during pregnancy. This enhancement includes improved pathogen detection, elevated phagocytic activity, and the activation of trained immunity, allowing the body to effectively defend against infections despite compromised adaptive immunity. For example, lactating laboratory rats presented an increased number of granulocytes and increased phagocytic activity in the blood, indicating an increase in innate immune functions to compensate for reduced adaptive immunity [24].

Monocytes function as cell phagocytes that defend against bacterial infections and are found in the circulatory system for short periods [32]. In humans, peripheral blood monocytes migrate out of circulation into areas of injury and help reduce inflammation and tissue restoration. During birth, for example, monocytes migrate to the cervix, myometrium, and endometrium [33, 34]. In general, the three genetic types presented a lower monocyte percentage at parturition. The lower monocyte percentage in the peripheral blood of adult RF and RFLP females, especially at parturition, could indicate the migration of monocytes from the bloodstream to reproductive tissues. This migration could indicate active tissue repair and inflammation regulation in these lines. This could indicate a change in the resource allocation of the new lines, allowing them to exhibit a distinct immune response during the stress of parturition. In contrast, the RF line presented a lower monocyte percentage at weaning than did the R and RFLP lines. For the RFLP line, the monocyte percentage significantly increased from the first AI to weaning. However, [7] reported the lowest monocyte count at the first AI.

The concentration of haptoglobin increases from the first AI to weaning. Haptoglobin is an acute-phase protein that modulates the immune response, such as minimising cellular damage during inflammation. An increase in the haptoglobin concentration is a nonspecific response related to inflammation that can be used indirectly to determine the health status of animals [35, 36]. The increase in this parameter could be due to the body’s effort to cope with all physiological and environmental changes and exposure to pathogens.

When comparing genetic types, R females had a lower percentage of total lymphocytes than RF and RFLP females did. In addition, the CD4+ percentage and CD4+ /CD8+ ratio for R females were lower than those for RFLP females. Since the CD4^+^/CD8^+^ ratio predicts immune regeneration, a lower ratio suggests that the immune system is less equipped to recover from infections [37]. In this particular study, the R line has the lowest ratio, the RF line has intermediate values, and the RFLP line has the highest values. The lower leukocyte percentage could account for the significant decrease in the percentage of females from the R line, which became significantly lower than that of the RFLP line by the second parturition. Kits from the R line also presented a lower percentage of T lymphocytes, CD4^+ cells^, and CD25^+ cells^ and a lower percentage of heterophil phagocytosis. Notably, the R line had higher monocyte and granulocyte percentages in adult females than both the RF and RFLP lines did. These results align with those of a previous study that compared the R line with two maternal lines, the long-living robust LP line and the hyper prolific H line [5]. In that study, the authors reported that the R line generally had lower leukocyte counts than monocytes and granulocytes did compared with the LP line [5].

Furthermore, in a study using two different generations from the R line, the authors suggested that a low leukocyte count could be a characteristic pattern exhibited by this genetic type [7]. Therefore, owing to the similarity of the results obtained in the present study, a low leukocyte percentage would indicate a normal health status for the R line and could reflect a strategy of immune tolerance. In this case, immune tolerance could be less energetically expensive and damaging to the host than a strong immune response [38]. In contrast, a high leukocyte percentage of the R line could be a sign of infection or disease.

Although R animals present lower leukocyte counts than other genetic types do, this might not necessarily translate to a worse immune status in the basal state [4, 7]. However, compared with other genetic types, with LP, for example, R animals are considered less robust [4]. We hypothesise that creating the RFLP line by introducing LP genetics might have changed the acquisition and allocation strategy related to the immune status [8]. The patterns previously observed showed that LP animals do not prioritise a specific life function. Instead, their enhanced capacity to acquire and allocate resources allows more effective distribution among different life functions and inhibits excess mobilisation of body reserves [39]. Furthermore, a different study comparing the LP line with a line selected for litter size (V) revealed that LP animals could cope better under heat stress challenge [40]. These strategies might have positively influenced the ability of the RFLP line to face environmental constraints and initiate an immune response.

The positive phenotypic correlation between the percentages of some leukocyte populations of females and kits at weaning suggests a possible maternal contribution to the innate and adaptative immunity of the offspring. These results align with those of a previous study in pigs, where most immune traits studied were moderately to highly heritable [41]. In this study, we observed that approximately 41% of the phenotypic variability of the CD4^+^/CD8^+^ ratio in weaned rabbits could be explained by this same ratio in their mothers. This ratio is a biomarker of the immune system’s ability to recover from infections [37], which could explain why RFLP females with higher CD4^+^/CD8^+^ ratios have litters with higher survival rates after weaning [9]. These results offer a promising perspective on the use of immune status in female rabbits to improve overall farm health [20].

These results demonstrated that leukocyte populations fluctuate during the productive cycle of female rabbits. Our findings indicate that these oscillations differ depending on the genetic type and moment of the reproductive cycle. However, the interaction between genetic type and physiological state does not appear to significantly alter most white blood cell populations. Previous work has shown that the introduction of LP genetics in the RFLP line appears to alter resource acquisition and allocation strategies, and this study seems to show that it potentially enhances the ability of the line to face environmental constraints and produce an effective immune response. Furthermore, female rabbits’ immune profiles may impact their immune status, highlighting potential maternal‒offspring interactions influencing immune parameters.

Finally, our results suggest that crossbreeding paternal rabbit lines with a longevity-focused maternal line enhances immune resilience, potentially improving animal health.

## Data Availability

The data that support these findings have not been deposited in an official repository.
